# Diminishing return for increased Mappability with longer sequencing reads: implications of the *k*-mer distributions in the human genome

**DOI:** 10.1186/1471-2105-15-2

**Published:** 2014-01-03

**Authors:** Wentian Li, Jan Freudenberg, Pedro Miramontes

**Affiliations:** 1 The Robert S. Boas Center for Genomics and Human Genetic, The Feinstein Institute for Medical Research, North Shore LIJ Health System, 350 Community Drive, Manhasset, USA; 2 Departamento de Matemáticas, Facultad de Ciencias, Universidad Nacional Autónoma de México, Circuito Exterior, Ciudad Universitaria, 04510 DF México, México

**Keywords:** Next-generation sequencing, Read alignment, Repeat sequences, Genome redundancy, Long-tail distribution, *k*-mers

## Abstract

**Background:**

The amount of non-unique sequence (non-singletons) in a genome directly affects the difficulty of read alignment to a reference assembly for high throughput-sequencing data. Although a longer read is more likely to be uniquely mapped to the reference genome, a quantitative analysis of the influence of read lengths on mappability has been lacking. To address this question, we evaluate the *k*-mer distribution of the human reference genome. The *k*-mer frequency is determined for *k* ranging from 20 bp to 1000 bp.

**Results:**

We observe that the proportion of non-singletons *k*-mers decreases slowly with increasing *k*, and can be fitted by piecewise power-law functions with different exponents at different ranges of *k*. A slower decay at greater values for *k* indicates more limited gains in mappability for read lengths between 200 bp and 1000 bp. The frequency distributions of *k*-mers exhibit long tails with a power-law-like trend, and rank frequency plots exhibit a concave Zipf’s curve. The most frequent 1000-mers comprise 172 regions, which include four large stretches on chromosomes 1 and X, containing genes of biomedical relevance. Comparison with other databases indicates that the 172 regions can be broadly classified into two types: those containing LINE transposable elements and those containing segmental duplications.

**Conclusion:**

Read mappability as measured by the proportion of singletons increases steadily up to the length scale around 200 bp. When read length increases above 200 bp, smaller gains in mappability are expected. Moreover, the proportion of non-singletons decreases with read lengths much slower than linear. Even a read length of 1000 bp would not allow the unique alignment of reads for many coding regions of human genes. A mix of techniques will be needed for efficiently producing high-quality data that cover the complete human genome.

## Background

Many applications of next-generation-sequencing (NGS) in human genetic and medical studies depend on the ability to uniquely align DNA reads to the human reference genome [1-6]. This, in turn, is related to the level of redundancy caused by repetitive sequences in the human genome, well known from the earlier human whole-genome shotgun sequencing [7,8], and the read length *k*. When the read length *k* is too short, it is theoretically impossible to have a reference sequence with size comparable to the human genome that does not contain any repeats of *k* bases. It has been shown using graph theory that the longest DNA sequences avoiding any repeats of *k*-mers can be constructed by packing all unique *k*-mers shifting one position at the time [9]. The number of different *k*-mer types is 4^*k*^/2 (*k* odd) or (4^*k*^+2^*k*^)/ 2 (*k* even) if both a subsequence and its reverse complement are considered to belong to the same *k*-mer type. Solving 4^*k*^/2≈3×10^9^ leads to the conclusion that read length *k* must be at least greater than 17 for all reads to be uniquely alignable to a hypothetical reference sequence that has the size of the human genome.

However, in reality the human genome did not evolve by a first principle to be consistently compact and incompressible. Redundant sequences in the human genome have resulted from duplication, insertion of transposable elements, and tandem repeats due to replication slippage, and more than half of the human genome can be traced to repetitive transposable elements. Although locally duplicated sequences can be deleterious [10] or disease-causing [11], a certain level of redundancy is a requirement for biological novelty and adaptation [12-14]. For higher eukaryotes, a slower removal of the deleterious repeats due to low mutation rates and smaller population sizes [15] lead to a higher level of genome-wide redundancy. This in turns may lead to more protein sequences with internal repeats and perhaps new fold or new functions such as the case for connection tissue, cytoskeletal, and muscle proteins [16].

Therefore, *k*=17 is a very unrealistic estimation of the minimal read length required for a perfectly successful NGS reads alignment. Accordingly, NGS technologies utilize reads with various larger lengths: *k*=70 for *Complete Genomics*, 35∼85 for *ABI SOLiD*, 75∼150 pair-end for *Illumina HiSeq*, 400 for *Ion Torrent PGM*, 450∼600 for *Roche 454 GS FLX Titanium XLR70*, etc. [17]. Currently, the technology is pushing towards read lengths of *k*=1000 (e.g., *Roche 454 GS FLX Titanium XL+*) or even *k*=10000 [18,19]. Needless to say, the longer the read length, the higher the chance that reads can be aligned to the reference genome. Ultimately, high quality genomes will be obtained by a mix of technologies. To find this optimal mixture, a quantitative understanding of the repeat structure of the human genome is required.

Our analysis of the repeat structure is different from some earlier investigations of read mappability [3,5]. In these studies, the actual reads from the current sequencing technology are used. There are two shortcomings in these approaches: (i) it is impossible to extrapolate the result to read lengths which is beyond the current technology; (ii) a certain proportion of reads are never mappable because the corresponding regions in the reference genome are not finished. Using the existing reference genome makes it possible to treat *k*-mers as hypothetic reads whose length *k* can be as long as possible, and unfinished regions can be excluded from the analysis.

In this paper we quantitatively address the question how alignment improves for greater read length. To this end, we artificially cut the human reference genome into overlapping *k*-windows (*k*-mers, *k*-tuples, or *k*-gram [20]), each considered to be possible a “read”, and count the number of appearances (or “tokens”, borrowing a terminology from linguistics [21]) of each *k*-mer type across the full reference sequence. Those *k*-mer types that appear in the genome only once (*f*=1) are labeled singletons, and the remainder (*f*>1) are non-singletons. Intuitively, the percentage of non-singleton reads is expected to decrease with increasing read length *k*. Obtaining the functional form of this decay enables us to predict the percentage of difficult-to-align reads for longer read lengths.

These seemingly simple calculations already encounter a “big data” problem on a regular-sized computer. In particular, storing counts in a hash table requires large amount of RAM. Suppose a *k*-mer needs K byte to store (e.g. *K*=*k*/4), a hash table to count all *k*-mers in the human genome would require 3*K* GByte RAM, which quickly becomes implausible when *k* is greater than 100. Using a solution that is similar to other applications where the hard disk [22-24] or computing time [25] is traded with RAM, we use a new public-domain program DSK which utilizes the less expensive hard disk and longer CPU time to compensate a lack of RAM [26]. Other efficient *k*-mer count procedures have been proposed in [27-29].

The mathematical relationship between the fraction of non-singleton *k*-mers and *k* predicts the fraction of putative reads that can be mapped uniquely. Another statistic of interest is the distribution of *k*-mer frequencies when *k* is fixed at a given value. This distribution has a head and a tail, a head for low frequency *k*-mers (including singletons), and a tail for high frequency *k*-mers. In the situation when these distributions exhibit long-tails [30] and power-law-like trends [31], thus fitting a straight line in log-log scale, the head end is best characterized by the frequency distribution [21], whereas the tail end is better characterized by the rank-frequency distribution commonly related to Zipf’s law in quantitative linguistics [32]. Our analysis of these distributions provides information on the level of redundancy in the human genome at various scales.

The identification of regions in the human genome that cannot be uniquely mapped by reads (which can be called “non-uniqueome” following the term “uniqueome” used in [3]) is important in any NGS-based studies. These regions may contribute the most the false-positive and false-negative variant callings. These may also be hotspots for structural variations such as copy-number-variation [33,34]. We will specifically examine the location of some of these regions at the *k*=1000 level.

## Methods

### Genome sequence data

The human reference genome GRCh37 (hg19) was downloaded from UCSC’s Genome Browser (http://genome.ucsc.edu/). The intermittent strings of N’s (marking unfinished basepairs that cannot be sequenced with the applied technology [35]) are used to partition the 22 autosomes and 2 sex chromosomes into 322 subsequences, and *k*-mers overlapping two chromosome partitions are not allowed.

For an additional analysis on repeat-filtered sequences, strings of lowercase letters in the reference genome (which mark repetitive sequences identified by the RepeatMasker program, http://www.repeatmasker.org/) are used to partition the genome into 3,456,905 subsequences with all transposable elements removed.

We further use the database *Dfam* version 1.2 (May 2013) (http://dfam.janelia.org/) [36] to annotate genomic regions by repeat sequences. *Dfam* contains the genomic locations of more than a thousand (1132) of transposable elements (TE) subfamily types. A hit is recorded whenever our genomic region overlaps with a TE. *Dfam* also provides information on tandem repeats by the program Tandem Repeat Finder [37].

Segmental duplication annotation of the human genome, which is either based on unusually high read coverage of whole-genome shotgun sequence segments from the Celera Genomics [38], or by a self-alignment by BLAST [39] on the RepeatMasker filtered genome (“fuguization”) [40,41], is obtained from the Segmental Dups track (“Duplications of > 1000 bases of non-RepeatMasker sequence”) at Genome Browser (http://genome.ucsc.edu/cgi-bin/hgTrackUi?g=genomicSuperDups).

### Counting *k*-mers

A *k*-mer type includes both the direct and the reverse complement substring; AAGC/GCTT is an example of such a 4-mer type. We use a state-of-art *k*-mer counting program DSK [26] (http://minia.genouest.org/dsk/), version 1.5031 (March 26, 2013). Most of the DSK calculations were carried out on a Linux computer with 48 GByte RAM and around 900 GByte disk space, except a calculation at *k*=1000 which was run on another Linux computer with the same RAM but 30 TByte of disk space. The parameter setting of DSK was determined by a trial-and-error process. The output of the DSK program consists of a list of *k*-mers. The BLAT program from UCSC’s Genome Browser is used to map frequent *k*-mers back to the reference genome.

### Frequency distribution, rank frequency plot, and data fitting

Suppose a *k*-mer type appears in the genome *f* times (*f* is frequency, or copy number); frequency distribution (FD) is the number of *k*-mer types with frequency *f*. Individual *k*-mer types can be ranked by their *f*, highest *f* ranks number 1, second highest *f* ranks number 2, etc. The ranked *f*’s of *k*-mer types as a function of rank *r* is the rank-frequency distribution (RFD).

The functions used here in fitting the RFD can all be expressed as linear regression, include Weibull function: log(*f*)∼ log(log((max(*r*)+1)/*r*)) [42]; quadratic logarithmic: log(*f*)∼ log(*r*)+(log*r*)^2^[43]; and reverse Beta: log(*r*)∼ log(*f*)+ log(max(*f*)+1-*f*). The latter function is derived from the Beta rank function [44-46] by reversing the *f* and *r*. All linear regressions are carried out by the *R* function *lm* (http://www.r-project.org/).

## Results

### Percentage of non-singleton reads vs. read length: piece-wise power-law function

**Figure 1:**
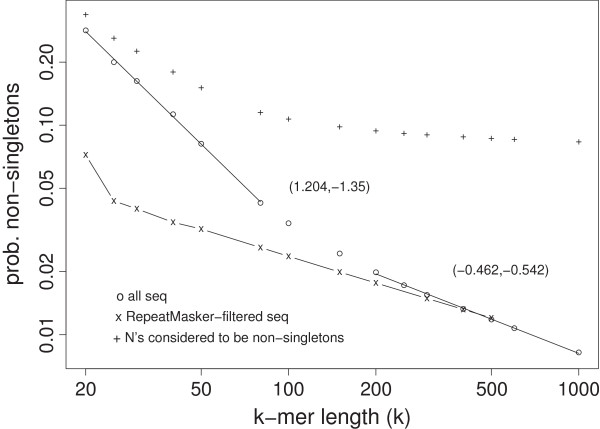
**Proportion of non-singleton*k*-mers/tokens in the human genome (24 chromosomes) as a function of*k* (in log-log scale).** Circles (o) show the results for all finished basepairs, whereas crosses (x) for the result from RepeatMasker-filtered sequences. Pluses (+) are results when unfinished sequences (234 Mbase) are included as non-singletons.

In Figure 1 we show the percentage of non-singleton reads/tokens (*p*_*ns*_) as a function of *k*-mer length *k* in log-log scale. The *p*_*ns*_ is 28.35% at *k*=20, 8.16% at *k*=50, 4.26% at *k*=80, 3.40% at *k*=100, 2.44% at *k*=150, 1.33% at *k*=400, 1.18% at *k*=500, and 0.82% at *k*=1000. If *k* is shorter than the “shortest unique substring” length, which is 11 in the human genome [47], singletons do not exist (i.e., *p*_*ns*_=100*%*).

Visual inspection of the trend suggests the use of piecewise power-law function in fitting the data. We fit the points in *k*=20-80 and *k*=200-1000 ranges separately by linear regressions in the log-log scale: log10*p*_*ns*_=*a*+*b* log10*k* (or log*p*_*ns*_∼ log*k*). The fitted $\left(â,\hat{b}\right)$ is (1.58366, -1.5478) and (-0.4371, -0.5495) for the two segments, equivalent to *p*_*ns*_=38.34/*k*^1.548^ and *p*_*ns*_=0.365/*k*^0.55^. The steep decay in the first segment shows a stronger increase of the amount of uniquely mappable sequences with read length, which implies that obtaining read lengths of at least around 100 is more cost-efficient with respect to reducing the amount of non-mappable reads. Of course, longer reads have extra benefits such as more robust alignments in the presence of polymorphisms or the ability to determine the length of longer repeat polymorphisms. The power-law function also indicates that the reduction of non-specific, difficult-to-align reads with longer read length is not linear.

If we assume our fitting function can be extrapolated to larger *k*’s for which a direct analysis of *k*-mer frequencies is restricted by computational constraints, the proportion of non-singleton reads can be predicted. For example, this leads to the prediction of a 0.2% non-singleton rate at the 10kb read length.

It is known that repetitive sequences create considerable obstacle in NGS alignment [48]. Though TE’s may exhibit subtle correlation with functional units in the genome [49], it is generally assumed that their biological role is indirect. Accordingly, we also looked at the non-singleton *k*-mer percentages in RepeatMasker filtered sequences (Figure 1). As expected, the percentage of uniquely mappable sequence is much higher than in the all-inclusive sequence for short *k*-mers (e.g. *k*<100). Interestingly, the differences between the two disappear for longer *k*-mers (e.g. *k*=500). A note of caution is that 89% of these RepeatMasker-filtered subsequences are shorter than 1kb, making the statistics less reliable at longer *k*’s.

### Maximum *k*-mer frequency decreases with *k* slowly

**Figure 2:**
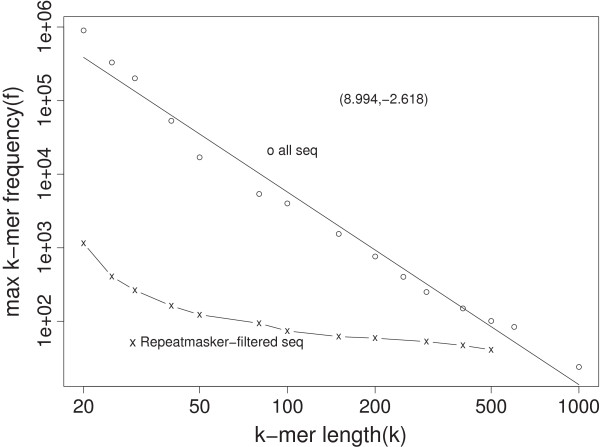
**Maximum frequencies of*k*-mers as a function of*k* (in log-log scale).** Circles (o) show the results for all finished bases, whereas crosses (x) for the result from RepeatMasker-filtered bases.

Another measure of the level of redundancy at length scale *k* is the maximum frequency (max(*f*)) of *k*-mer types. For example, base A/T homopolymers of length 20 appear most often with 898,647 copies; at *k*=400, AT repeats have more copy numbers (*f*=150) than other 400-mers; the max(*f*) for *k*=1000 is equal to 24 for a sequence which is not filtered by the RepeatMasker. The max(*f*) as a function of *k* is shown in Figure 2 in log-log scale.

For RepeatMasker-filtered sequences, max(*f*) quickly decays below 100 and then falls only slowly, indicating that RepeatMasker usually finds shorter repeats. At *k*= 200–500, the *k*-mer with the max(*f*) ∼ 50 is a lowcomplexity sequence, with internal repeats of GGGGGGAACAGCGACAC/GTGTCCGCTGTTCCCCCC. Despite its high prevalence, this low-complexity sequence is not masked by RepeatMasker in the human reference genome.

Fitting the linear regression model log10 max(*f*)=*a*+*b* log10*k* (or log max(*f*)∼ log*k*) leads to (*a*,*b*)= (8.99, -2.62). Extrapolating this regression to longer *k*’s predicts that at *k*=2724, max(*f*) = 1. This prediction should be viewed with caution as max(*f*) is mainly determined by “outlier” events thus un-reproducible in principle, and the linear function in Figure 2 does not fit the data perfectly. Any extrapolation, exemplified by both Figure 1 and Figure 2, is based on the assumption that the fitted function in the observed range will continue as the same outside the range. There is no guarantee that this assumption is true in the present case.

### Frequency distributions at fixed k values exhibit power-law-like trend

**Figure 3:**
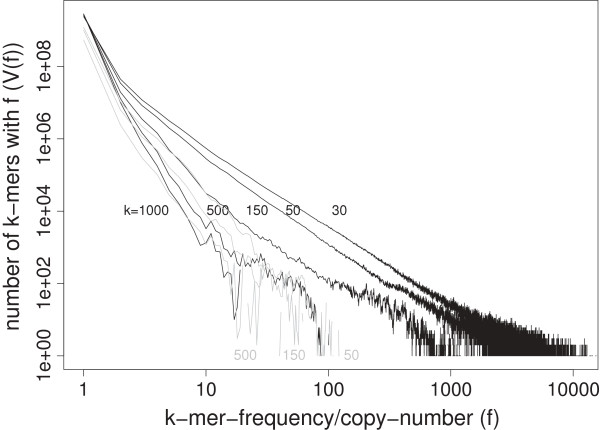
**Frequency distributions of*k*-mers at*k* = 30, 50, 150, 500, and 1000 (in log-log scale).** The distributions for *k*-mers in repeat-filtered sequences at *k*=50, 150, 500 are shown in grey lines.

The frequency distribution (FD) describes the distribution of *k*-mer types according their copy numbers in the genome. When plotted in log-log scale, low-frequency *k*-mer types and the less redundant portion of the sequence are highlighted. Figure 3 shows five FDs at *k*=30, 50, 150, 500, and 1000 in log-log scale. The FDs at *k*=30 and 50 span a wider frequency range, and the power-law trend is obvious.

A similar FD for *k*=40 in human genome was shown in [50,51], and a slope of -2.3 in linear regression (in log-log scale) in the *f*= 3–500 range was reported. When we fit the *k*=50 FD by linear regression in log-log scale, a very similar fitting slope value is obtained (-2.38, for *f*= 3-200). However, it is clear from Figure 3 that the slopes are steeper for *k*=150 (-2.7 for *f*= 2-100), *k*=500 (-3.5 for *f*= 2-40), and *k*=1000 (-5.3 for *f*= 2–19, or -5.9 from *f*= 2-9), indicating that the slope is not a universal parameter.

From the short read alignment perspective, the long tail at the high copy-numbers shows that many sequences cannot be uniquely mapped at smaller *k* values (e.g. *k*=30, 50). However, the tail is much shortened at *k*=1000. As expected, the tail for RepeatMasker-filtered sequences at various *k* values are much shorter (Figure 3, grey lines).

### Rank-frequency distributions at fixed k values mostly follows a concave curve in log-log scale

**Figure 4:**
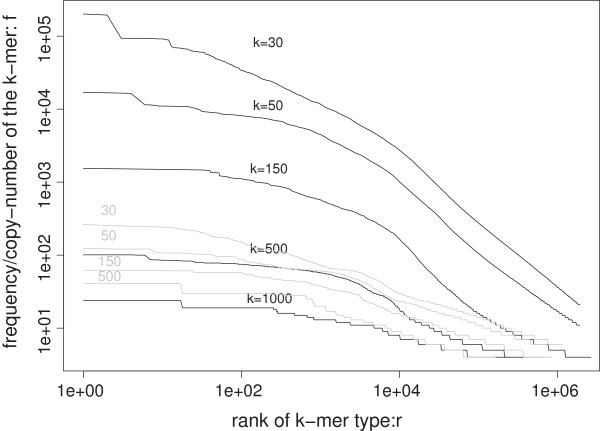
**Rank-frequency distributions for*k*-mers at*k* = 30, 50, 150, 500, and 1000 (in log-log scale).** The corresponding rank-frequency distributions for RepeatMasker-filtered sequences at *k*=30, 50, 150, 500 are shown in grey lines.

Although a rank-frequency distribution (RFD) can be converted to cumulative FD [42], in log-log scale, it zooms into the high-frequency tail of the frequency distribution. Figure 4 shows five RFD at *k*’s from 30 to 1000. While the RFD at *k*=30 may maintain a power-law or piecewise power-law trend, those at larger *k* values become more concave. This concave Zipf’s curve is commonly observed in city size distributions [52,53].

**Figure 5:**
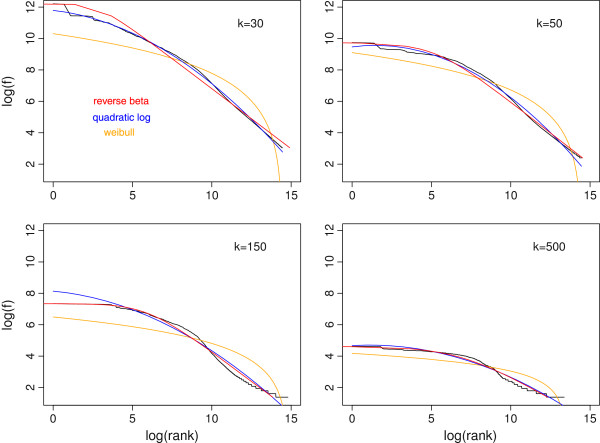
**Fitting rank-frequency distribution of*k*-mers at k = 30, 50, 150, 500 using three functions.** Red: quadratic logarithmic (log*f*∼ log(*r*)+ log((*r*))^2^, *f*: frequency of a *k*-mer type, *r*: rank of a *k*-mer type, and the ∼ symbol represents linear regression); blue: reverse Beta rank function (log(*r*)∼ log(*f*)+ log(*max*(*f*)+1-*f*)); Orange: Weibull function (log(*f*)∼ log(log((*max*(*r*)+1)/*r*))).

For RFDs deviating from the Zipf’s law, functions with two parameters may be used to account for the concave or convex shape of the curve in log-log scale [42]. We found that the quadratic logarithmic function, but not the Weibull function, fits the RFDs well (Figure 5). The Beta rank function usually exhibit “S” shapes [45], whereas the RFD in Figure 4 shows a “Z” shape. This motivated us to use a novel reverse Beta function to fit the data (Figure 5). The “Z” shaped log-log RFD means that if the power-law function is the default functional relationship between frequency and rank, frequencies of the intermediately-ranked *k*-mers decrease faster than the two tails. The “S” shaped log-log RFD implies the opposite.

### Mapping ***f≥10*** 1000-mer to the reference genome

For *k*=1000, there are 6107 *k*-mer types with frequency *f* larger or equal to 10. Due to the fact that these are overlapping *k*-mers, they are mapped to only 172 chromosomal regions, each of a few kb (the 172 locations, number of high-frequency 1000-mers, and the distance from the left-neighboring chromosome regions are included in Additional file 1: Table S1).

A total of 70 out of these 172 regions (or 40%) are clustered in four larger stretches on chromosomes 1 and X and contain long tandem repeats (60, 70 kbase on chromosome 1q21.1, 1q21.2, and 41, 56 kbases on Xq23, Xq24). The two stretches on chromosome 1 contain copies of the neuroblastoma breakpoint family genes (*NBPF*) [54-56]. The Xq24 region contains cancer/testis antigen family genes (*CT47A*) [57,58], whereas the Xq23 region has no genes, but contains the macrosatellite *DXZ4*[59-61] which exhibits periodic appearance of other functional elements, such as H3K27Ac or H3K4me2 [62] histone modification marks.

Besides these long stretches, 39 out of 172 regions (or 23%) overlap with 34 genes: *ZNF3850, EPHA3, COL6A6, CD38, KCNIP4, FRAS1, ANTXR2, HSD17B11, FAM190A, DKK2, FBXL7, AK123816, FAM153A, FAM65B, LAMA2, MYCT1, NOD1, TPST1, PSD3, KCNB2, NR4A3, C9orf171, CACNA1B, DLG2, CCDC67, UACA, HOMER2, SMG1, CDH13, PRKCA, LILRA2, TTC28, MTMR8*, and *SLC25A43*. Obtaining high quality data on genetic variants in these genes is therefore likely to remain a challenge even with longer reads.

The distribution of transposable elements in the 172 regions is analyzed using the *Dfam* database. Interestingly, 1q21.1, 1q21.2, Xq23 regions discussed above do not overlap with any transposable elements. The Xq24 region contains a subfamily of Alu, AluSc8 (length ∼ 304, with mismatch-included copy number in the human genome ∼ 24000). Outside the four long stretches of genomic regions, however, almost all overlap with LINE-1 retrotransposons [63] (98/102, or 96%; 98/172, or 57%). Among these, the dominant LINE-1 subfamily is L1P1_orf2 (84/102, or 82%; 84/172 or 49%). The length of L1P1_orf2 is roughly 2174, and its mismatch-included copy number in the human genome is more than 16000.

Other LINE-1 subfamilies overlapping these regions include L1P1_5end, L1M2_5end, L1PA2_3end, and L1ME3G_3end. Three regions also overlap with a DNA transposon, Tigger3d. All transposable element information in these regions are listed in the Additional file 1: Table S1. Additional file 1: Table S1 also shows the tandem repeats result, such as TG-, AC-, or TTTA-repeat. Unlike transposable elements, these tandem repeats comprise a very small proportion of the region.

The Segmental Duplications Track in the Genome Browser provides repeat information that is different from the transposable elements. These repeats are usually large (> 1-15kb), and information is obtained either from the whole-genome shotgun sequencing reads, independent from the reference genome; or from the reference genome itself by self-alignment. We have listed overlapping information between our 172 regions and those in the Segmental Duplications Track in the Additional file 1: Table S1. Reassuringly, the four large regions on chromosomes 1 and X overlap with the previously identified segmentally duplicated regions, even though the methodology of the two approaches are very different.

By inspecting the Additional file 1: Table S1, it can be seen that the 172 regions either contain LINE transposable elements or overlap with the segmental duplication track. The large stretch on Xq24 overlaps with both segmental duplication track and transposable elements. However, the transposable element contained is the Alu element, which is a SINE instead of LINE. Possible connections between segmental duplication and Alu elements have been discussed before [64], and it is possible that the Alu element appeared in this region before the onset of duplication.

## Discussion

### Long *k*-mers in the reference genome as surrogate for sequencing reads

The *k*-mer distribution has many application in sequence analysis, such as measuring similarity between two genomes [65], correcting sequencing error [66], finding repeat structures [67], determining the feasibility of gene patents [68]. In many applications, only short *k*-mers are considered to be relevant, such as *k*=6 [69], *k*≤7 [70], *k*=8 [71], *k*=11 [72]. This paper essentially uses long *k*-mers taken from the reference genome as surrogate for reads from future NGS technologies. Computationally speaking, counting long *k*-mers is more challenging and we are not aware of any prior publications on the long *k*-mer distributions in the human genome for *k* as long as 1000.

As compared to other papers on mappability of genome sequencing reads [3,5], our more theoretical approach has the advantage of being able to discuss long reads (e.g. *k*=1000) where such data is not available from the current NGS technology. Our approach also separates the two causes of poor mappability: one due to the unfinished sequence in the reference genome and another due to the redundancy in the finished sequences. The unfinished bases are mainly located in the centromeres, short arms of acrocentric chromosomes and other heterochromatic regions, and rich in repetitive sequences. If we always treat this unfinished sequences (total 234 Mbases) to be non-singletons regardless of *k*, *p*_*ns*_ would flatten out around 0.1 (see Figure 1).

### A baseline knowledge of redundancy of the human genome at length *k* level

Figures 1, 2 and 3 provides a baseline knowledge of the redundancy of the human genome at the *k*-mer level. Our results give a quantitative description of the effect of read length *k* on the mappability of reads from the finished region of the human genome.

Reference assembly is easier than *de novo* assembly, and our approach does not directly apply to *de novo* sequencing “assemblability”. However mappability and assemblability are closely related, as repetitive sequences cause problems in both situations [73]. The current *de novo* assemblies still do not perform consistently [74,75] and a quantitative assessment of the impact of repetitive sequences on reference assembly could be a useful piece of information for *de novo* assembly as well. Note that some discussion on *k*-mer-based assembly actually refers to *k*^′^-mer (*k*^′^<<*k*) [76,77].

### Highly redundant regions at *k* = 1000 level and copy-number-variation regions

The chromosome 1 and X regions which we have identified by showing at least 10 copy numbers of 1000-mers are discussed in the literature as regions with common copy-number-variations (CNV). CNVs in the 1q21.1 region, if not *NBPF*-specific, have been linked to congenital cardiac defects [78-80], autism [81,82], mental retardation [83], head size abnormalities [84], schizophrenia [85,86], and neuroblastoma [87]. With so many abnormalities mapped to this region, these are collectively called the chromosome 1q21.1 duplication syndrome in the Online Mendelian Inheritance in Man (OMIM 612475).

The Xq23 region, if not macrosatellite *DXZ4* specific, has been identified as likely CNV regions linked to developmental and behavioral problems [88]. Chromatin configuration at *DXZ4* region is reported to differ between male melanoma cells and normal skin cells [89]. The Xq24 and the *CT47A* gene are listed as a region of structural variants associated with intellectual disability [90] and mental retardation [91].

A well-known mechanism for CNV formation is non-allelic homologous recombinations (NAHR) between repetitive elements [92]. More copies of a repetitive sequence give more opportunities that NAHR could occur, resulting in a natural connection between repetitive sequences and CNV. The fact that simple counting of 1000-mer frequencies leads to CNV regions with medical implications indicates that understanding the *k*-mer distribution is an important part of genomic analyses.

Although the four highlighted large regions also appear in the Segmental Duplication track for >1000 bp RepeatMasker-filtered sequences in the UCSC Genome Browser, the two methodologies are somewhat different. Here, we use the reference genome as starting point, length scale is upper-limited at 1000 bp, zero-mismatch, and high copy numbers (≥10). In SegDup track, the reference may or may not be used (in the latter case, raw reads are the starting point), length scale is lower-limited at one or few kbs, mismatches are allowed, and low copy number (e.g. 2) is allowed. From this may lead to the development of strategy where our approach can be used to check the consistency of the reference genome with raw read data.

### Discussions of extensions to a next-generation-sequencing data

In a realistic setting of NGS, there are sequencing errors and single-nucleotide polymorphisms (SNP); alignment to the reference genome may allow mismatches; and there is a wide adoption of paired-end/mate-pair strategy [93-96]. It is a daunting challenge to provide a definitive answer under these situations [4] for long *k*-mer lengths such as *k*=1000. Some concepts in this paper, e.g., the *k*-mer frequency distribution in Figure 3, cannot be used if mismatches are considered.

We can however speculate about some consequences when practical complications are introduced. Suppose a DNA fragment (of length *k*) is split into two ends (of length *k*^′^<*k*/2 each) which are to be sequenced, and an insert (of length *k*-2*k*^′^). At *k*^′^=*k*/2, one is essentially sequencing the whole DNA fragment, and aligning two *k*^′^-mers next to each other is equivalent to aligning a 2*k*^′^-mer. The result in Figure 1 implies that the proportion of non-mappable reads/tokens decreases with *k*^′^ as 1/(2*k*^′^)^*b*^. When *k*≪2*k*^′^, aligning two paired-end *k*^′^-mers is more likely to be unique than when the two *k*^′^-mers are next to each other, as the correlation between two *k*^′^-mers decrease with distance [97]. We may speculate that the proportion of non-uniquely-mapped reads as a function of *k*^′^ and *k* is: ∼*f*(*k*-2*k*^′^)/(2*k*^′^)^*b*^, where the unknown function *f*(*k*-2*k*^′^) is 1 if *k*=2*k*^′^, and decreases with *k*-2*k*^′^.

There have been recent attempts to fill in the sequence of inserts between two ends in the pair-end strategy [98-101]. A typical example would consider a segment length *k* of 600-800 bp, and read length *k*^′^ of 100 bp [101]. We then can consider the best scenario that the sequence of the whole segment of length *k* can be determined. This will merely shift the length scale from the two times the read length (2*k*^′^) to the segment length (*k*), and all our results still apply.

The effect of sequencing errors, single-nucleotide polymorphism, alignment allowing mismatches, can be discussed in the framework of *k*-mer space (with reverse complement). The observed *k*-mers in the human genome consist of a subspace of the *k*-mer space, and a link between two *k*-mers is established when the Hamming distance between the two is 1. Sequencing errors and polymorphisms either generate a new *k*-mer in this subspace, or move along a link to a previously existing *k*-mer. If new *k*-mers are generated, links between *k*-mers will be recalculated. One can argue that sequencing error and polymorphism would have less impact if the error/mutation does not lead to the creation of a new *k*-mer, or, even when a new *k*-mer is created, if the new *k*-mer does not have new links to other *k*-mers. In the case where sequencing errors and polymorphisms generate two or more mutations, links between *k*-mers with both 1- and 2-Hamming distances should be considered. The framework of discussion is similar, though more complicated.

### Long-tails and the regime of diminishing return of longer reads

Our analysis shows that all distributions discussed in this paper are better viewed in log-log scale, proving the existence of power-law distributions or long-tails. This has been observed in the past for other genomic distributions, such as correlation function [97,102-104], power spectrum of base composition [105-108], frequency distribution of gene or protein family size [109-112], sizes of ultraconserved regions [113], and in models with duplications [114-117]. Ongoing duplications increase the copy number geometrically, which explains the presence of long-tails.

A consequence of the long-tail in Figure 1 is that with increasing read (or *k*-mer) lengths, the proportion of reads that cannot be mapped to a unique genomic region (within the finished sequences) decreases as a power-law function, as compared to a linear or exponential function. Numerically, if not economically, this defines a regime of diminishing return. It is important to emphasize that we have only directly observed an diminishing return in the range of 200-1000 bp. This diminishing return may be extended further beyond 1kb, until it reaches a point of accelerating return if the read length is longer than the size of any segmental duplication region (which can be 200kb for gene-containing duplications [118]). The use of paired-end strategy usually does not increase the length scale by orders of magnitude, thus it may still be confined to the diminishing return regime. To assess the economic return with NGS technology with longer reads, other factors should be considered, such as the choice of less redundant target regions such as the exome [119], read length and sequencing error tradeoff, and the overall cost of longer-read sequencing.

## Conclusion

We have established that, up to 1000 bases, the mappability of reads decreases slower than linear with read length, when mappability is measured as the proportion of non-singletons in human reference genome. The slow decrease is similar to other observed long tail distributions in genomics. Anticipating that the highest-quality human genome sequences will be obtained by a combination of various technologies, the analysis of *k*-mer distribution at different scales is a prominent factor for determining how these technologies can be optimally combined. We also identified the most redundant 1000-mers in the human genome, which include the region responsible for the chromosome 1q21.1 duplication syndrome, as well as other regions which are rich in segmental duplication and macrosatellites.

## Availability of support data

The data set supporting the results of this article is included within the article and its additional file.

## Abbreviations

BLAT: BLAST like alignment tool; BLAST: Basic local alignment search tool; CPU: Central processing unit; CNV: Copy number vatiations; DSK: Disk streaming of kmers; DNA: Deoxyribo-nucleic acids; FD: Frequency distribution; GRCh37: Genome reference consortium human (build) 37; LINE: Long interspersed elements; NAHR: Non-allelic homologous recombinations; NGS: Next-generation sequencing; OMIM: Online Mendelian Inheritance in Man; RAM: Random-access memory; RFD: Rank-frequency distribution; SINE: Short interspersed elements; SNP: Single nucleotide polymorphism; TE: Transposable elements; UCSC: University of California at Santa Cruz.

## Competing interests

The authors declare that they have no competing interests.

## Authors’ contributions

WL conceived of the study and contribute to the analysis of the data. JF carried out the mapping of redundant 1000-mers to the reference genome. PM carried out the fitting of rank-frequency distribution. WL, JF, PM contribute to draft of the manuscript. All authors read and approved the final manuscript.

## Supplementary Material

Additional file 1 **The additional file includes the supplementary Table S1: 172 chromosome locations with high-frequency (*f*** ***≥* 10) 1000-mers.** Click here for file
