# Supplementary material for: Diminishing return for increased Mappability with longer sequencing reads: implications of the k-mer distributions in the human genome
Source: BMC Bioinformatics. 2014 Jan 3;15:2. doi: 10.1186/1471-2105-15-2 (PMC3927684; doi:10.1186/1471-2105-15-2)
Supplement: Additional file 1 — The additional file includes the supplementary Table S1: 172 chromosome locations with high-frequency ( f ≥ 10) 1000-mers. [file 1471-2105-15-2-S1.pdf]

# Supplementary material: Diminishing Return for Increased Mappability with Longer Sequencing Reads: Implications of the $k$-mer Distributions in the Human Genome

Wentian Li, an Freudenberg, Pedro Miramontes

## Supplementary material: Table S1

Chromosome locations of $k$=1000-mers with frequency $f \geq 10$ mapped to the human reference genome (GRCh37/hg19, Feb 2009). The eight columns are: 1. chromosome (23 for chromosome X); 2. starting position (in base); 3. ending position (in base); 4. chromosome band; 5. width of the region (in kbases); 6. number of 1000-mers mapped to this region; 7. spacing with the previous region (in base); 8. (if available) gene name; 9. (if available) transposable elements (TE) and tandem repeats (TR); 10. (if available) percentage of identity in segmental duplication.

The transposanle elements and tandem repeats information is obtained from the Dfam (*http://dfam.janelia.org/*). The segmental duplication information is obtained from the "Duplications of >1000 bases of non-repeatMasked sequence" track in the Genome Browser: (*http://genome.ucsc.edu/cgi-bin/hgTrackUi?g=genomicSuperDups*). If there is an orange bar overlapping the region, we write +99% in the Table S1, whether there is also a yellow/grey bar or not. If there is a yellow bar, we write +98%. If there is a grey/black bar but no yellow/orange bar, we write +90%.

| ch | start | end | ch_band | width(kb) | n_entity | gap(bp) | gene_name | TE/TR | %_in_DUPS |
|---|---|---|---|---|---|---|---|---|---|
| 1 | 56831364 | 56833043 | 1p32.2 | 1.679 | 390 | 56831364 | | L1P1_orf2 | |
| 1 | 84518510 | 84519720 | 1p31.1 | 1.210 | 162 | 27685467 | | L1P1_orf2 | |
| 1 | 84521988 | 84523534 | 1p31.1 | 1.546 | 333 | 2268 | | L1P1_orf2,L1M2_5end,L1P1_5end | |
| 1 | 144163689 | 144164940 | 1q21.1 | 1.251 | 253 | 59640155 | NBPF-family | | +98% |
| 1 | 144164949 | 144166172 | 1q21.1 | 1.223 | 156 | 9 | NBPF-family | | +98% |
| 1 | 144166529 | 144168209 | 1q21.1 | 1.680 | 682 | 357 | NBPF-family | TG-repeat | +98% |
| 1 | 144171267 | 144172868 | 1q21.1 | 1.601 | 603 | 3058 | NBPF-family | TG-repeat | +98% |
| 1 | 144174458 | 144177621 | 1q21.1 | 3.163 | 1238 | 1590 | NBPF-family | TG-repeat | +98% |
| 1 | 144178038 | 144179323 | 1q21.1 | 1.285 | 287 | 417 | NBPF-family | | +98% |
| 1 | 144179746 | 144180804 | 1q21.1 | 1.058 | 60 | 423 | NBPF-family | TG-repeat | +98% |
| 1 | 144180830 | 144184012 | 1q21.1 | 3.182 | 710 | 26 | NBPF-family | TG-repeat | +98% |
| 1 | 144190348 | 144193522 | 1q21.1 | 3.174 | 961 | 6336 | NBPF-family | TG-repeat | +98% |

| | | | | | | | | | |
|---|---|---|---|---|---|---|---|---|---|
| 1 | 144193538 | 144195088 | 1q21.1 | 1.550 | 552 | 16 | NBPF-family | TG-repeat | +98% |
| 1 | 144195507 | 144201554 | 1q21.1 | 6.047 | 1426 | 419 | NBPF-family | TG-repeat | +98% |
| 1 | 144203065 | 144204372 | 1q21.1 | 1.307 | 264 | 1511 | NBPF-family | TG-repeat | +98% |
| 1 | 144206755 | 144207807 | 1q21.1 | 1.052 | 54 | 2383 | NBPF-family | | +98% |
| 1 | 144207827 | 144209509 | 1q21.1 | 1.682 | 684 | 20 | NBPF-family | TG-repeat | +98% |
| 1 | 144211012 | 144214175 | 1q21.1 | 3.163 | 1238 | 1503 | NBPF-family | TG-repeat | +98% |
| 1 | 144214195 | 144215877 | 1q21.1 | 1.682 | 684 | 20 | NBPF-family | TG-repeat | +98% |
| 1 | 144217378 | 144220552 | 1q21.1 | 3.174 | 961 | 1501 | NBPF-family | TG-repeat | +98% |
| 1 | 144222542 | 144223699 | 1q21.1 | 1.157 | 154 | 1990 | NBPF-family | | +98% |
| 1 | 148255552 | 148256803 | 1q21.2 | 1.251 | 253 | 4031853 | NBPF-family | | +99% |
| 1 | 148257416 | 148258725 | 1q21.2 | 1.309 | 260 | 613 | NBPF-family | AC-repeat | +90% |
| 1 | 148260316 | 148261567 | 1q21.2 | 1.251 | 253 | 1591 | NBPF-family | | +98% |
| 1 | 148263509 | 148265059 | 1q21.2 | 1.550 | 552 | 1942 | NBPF-family | AC-repeat | +98% |
| 1 | 148265073 | 148268247 | 1q21.2 | 3.174 | 710 | 14 | NBPF-family | AC-repeat | +98% |
| 1 | 148268281 | 148269831 | 1q21.2 | 1.550 | 552 | 34 | NBPF-family | AC-repeat | +98% |
| 1 | 148271714 | 148273021 | 1q21.2 | 1.307 | 264 | 1883 | NBPF-family | AC-repeat | +98% |
| 1 | 148273051 | 148277793 | 1q21.2 | 4.742 | 1139 | 30 | NBPF-family | AC-repeat | +98% |
| 1 | 148278120 | 148280976 | 1q21.2 | 2.856 | 931 | 327 | NBPF-family | AC-repeat | +98% |
| 1 | 148282487 | 148284169 | 1q21.2 | 1.682 | 684 | 1511 | NBPF-family | AC-repeat | +98% |
| 1 | 148285680 | 148286965 | 1q21.2 | 1.285 | 287 | 1511 | NBPF-family | | +98% |
| 1 | 148287392 | 148288944 | 1q21.2 | 1.552 | 554 | 427 | NBPF-family | | +98% |
| 1 | 148288961 | 148291735 | 1q21.2 | 2.774 | 563 | 17 | NBPF-family | | +98% |
| 1 | 148292162 | 148293714 | 1q21.2 | 1.552 | 554 | 427 | NBPF-family | | +98% |
| 1 | 148293731 | 148296505 | 1q21.2 | 2.774 | 563 | 17 | NBPF-family | | +98% |
| 1 | 148296932 | 148298484 | 1q21.2 | 1.552 | 554 | 427 | NBPF-family | | +98% |
| 1 | 148298501 | 148301275 | 1q21.2 | 2.774 | 561 | 17 | NBPF-family | | +98% |
| 1 | 148301694 | 148303244 | 1q21.2 | 1.550 | 552 | 419 | NBPF-family | AC-repeat | +98% |
| 1 | 148303258 | 148306035 | 1q21.2 | 2.777 | 311 | 14 | NBPF-family | | +98% |
| 1 | 148308035 | 148309286 | 1q21.2 | 1.251 | 253 | 2000 | NBPF-family | | +98% |
| 1 | 148311228 | 148312780 | 1q21.2 | 1.552 | 554 | 1942 | NBPF-family | | +98% |
| 1 | 148312794 | 148315968 | 1q21.2 | 3.174 | 963 | 14 | NBPF-family | AC-repeat | +98% |
| 1 | 148317640 | 148318820 | 1q21.2 | 1.180 | 101 | 1672 | NBPF-family | | +98% |
| 1 | 148320748 | 148322300 | 1q21.2 | 1.552 | 554 | 1928 | NBPF-family | | +98% |
| 1 | 148322314 | 148325488 | 1q21.2 | 3.174 | 708 | 14 | NBPF-family | AC-repeat | +98% |
| 1 | 237186076 | 237187264 | 1q43 | 1.188 | 15 | 88860588 | | L1P1_orf2,L1M2_5end | |
| 1 | 247852722 | 247854662 | 1q44 | 1.940 | 370 | 10665458 | | L1P1_orf2 | |
| 2 | 4782957 | 4784039 | 2p25.2 | 1.082 | 68 | NA | | L1M2_5end,L1P1_orf2 | |

| | | | | | | | | | |
|---|---|---|---|---|---|---|---|---|---|
| 2 | 11138506 | 11139623 | 2p25 | 1.117 | 51 | 6354467 | | L1P1_orf2 | |
| 2 | 134968954 | 134970563 | 2q21.2 | 1.609 | 162 | 123829331 | | L1P1_orf2 | |
| 3 | 22092360 | 22093571 | 3p24.3 | 1.211 | 63 | NA | ZNF3850 | L1P1_5end | |
| 3 | 22096119 | 22097785 | 3p24.3 | 1.666 | 577 | 2548 | ZNF3850 | L1P1_orf2 | |
| 3 | 80925499 | 80926606 | 3p12.2 | 1.107 | 34 | 58827714 | | L1ME3G_3end,L1PA2_3end,Tigger3d | |
| 3 | 89513287 | 89515334 | 3p11.1 | 2.047 | 638 | 8586681 | EPHA3 | L1P1_orf2,L1M2_5end,L1P1_5end | |
| 3 | 108920256 | 108921439 | 3q13.13 | 1.183 | 93 | 19404922 | | L1M2_5end,L1P1_orf2 | |
| 3 | 130351657 | 130352915 | 3q22.1 | 1.258 | 260 | 21430218 | COL6A6 | L1P1_orf2 | |
| 3 | 137072367 | 137073449 | 3q22.3 | 1.082 | 84 | 6719452 | | L1PA2_3end | |
| 4 | 15845170 | 15846306 | 4p15.32 | 1.136 | 138 | NA | CD38 | L1P1_orf2 | |
| 4 | 21163013 | 21164348 | 4p15.31 | 1.335 | 237 | 5316707 | KCNIP4 | L1P1_orf2 | |
| 4 | 21165563 | 21167050 | 4p15.51 | 1.487 | 448 | 1215 | KCNIP4 | L1P1_orf2 | |
| 4 | 71194736 | 71195804 | 4q13.3 | 1.068 | 40 | 50027686 | | L1P1_5end,L1M2_5end | |
| 4 | 75644610 | 75646550 | 4q13.3 | 1.940 | 370 | 4448806 | | L1P1_orf2 | |
| 4 | 79271909 | 79273254 | 4q21.21 | 1.345 | 73 | 3625359 | FRAS1 | L1P1_orf2 | |
| 4 | 80863626 | 80864733 | 4q21.21 | 1.107 | 34 | 1590372 | ANTXR2 | L1P1_orf2 | +99% |
| 4 | 80890063 | 80891544 | 4q21.21 | 1.481 | 131 | 25330 | | L1P1_orf2 | |
| 4 | 88268648 | 88269936 | 4q22.1 | 1.288 | 210 | 7377104 | HSD17B11 | L1P1_orf2 | |
| 4 | 88270964 | 88272227 | 4q22.1 | 1.263 | 165 | 1028 | HSD17B11 | L1P1_orf2 | |
| 4 | 88272229 | 88273386 | 4q22.1 | 1.157 | 159 | 2 | HSD17B11 | L1P1_orf2,L1M2_5end | |
| 4 | 91600685 | 91602074 | 4q22.1 | 1.389 | 147 | 3327299 | CCSER1 | L1P1_orf2,L1M2_5end | |
| 4 | 108005014 | 108277809 | 4q25 | 272.795 | 128 | 16402940 | DKK2 | | |
| 4 | 137214667 | 137216333 | 4q26.3 | 1.666 | 577 | 28936858 | | L1P1_orf2 | |
| 4 | 137217568 | 137220031 | 4q26.3 | 2.463 | 776 | 1235 | | L1P1_orf2,L1M2_5end,L1P1_5end | |
| 4 | 139471548 | 139473214 | 4q26.3 | 1.666 | 577 | 2251517 | | L1P1_orf2 | |
| 5 | 10479 | 11579 | 5p15.33 | 1.100 | 60 | NA | | | |
| 5 | 15906794 | 15907818 | 5p15.1 | 1.024 | 26 | 15895215 | FBXL7 | L1P1_orf2 | |
| 5 | 57682240 | 57683559 | 5q11.2 | 1.319 | 49 | 41774422 | | L1P1_orf2 | |
| 5 | 103855313 | 103857622 | 5q21.2 | 2.309 | 547 | 46171754 | | L1M2_5end, L1P1_orf2 | |
| 5 | 108595761 | 108598576 | 5q21.3 | 2.815 | 772 | 4738139 | | L1P1_5end,L1M2_5end,L1P1_orf2 | |
| 5 | 152268410 | 152269605 | 5q33.1 | 1.195 | 120 | 43669834 | AK123816 | L1P1_orf2 | |
| 5 | 166395979 | 166397375 | 5q35.3 | 1.396 | 381 | 14126374 | | L1P1_orf2 | |
| 5 | 177201688 | 177203367 | 5q35.3 | 1.679 | 390 | 10804313 | FAM153A | L1P1_orf2 | +99% |
| 5 | 177203999 | 177205005 | 5q35.3 | 1.006 | 8 | 632 | FAM153A | L1P1_orf2 | +99% |
| 6 | 24814196 | 24815833 | 6p22.3 | 1.637 | 335 | NA | FAM65B | L1P1_orf2 | |
| 6 | 24816124 | 24817135 | 6p22.3 | 1.011 | 13 | 291 | FAM65B | L1M2_5end | |
| 6 | 86712093 | 86713844 | 6q14.3 | 1.751 | 383 | 61894958 | | L1P1_orf2, L1M2_5end | |

| | | | | | | | | |
|---|---|---|---|---|---|---|---|---|
| 6 | 129321768 | 129322865 | 6q22.33 | 1.097 | 99 | 42607924 | LAMA2 | L1P1_orf2 |
| 6 | 133342240 | 133343397 | 6q23.2 | 1.157 | 141 | 4019375 | | L1P1_orf2 |
| 6 | 133344551 | 133346228 | 6q23.2 | 1.677 | 285 | 1154 | | L1P1_orf2, L1M2_5end |
| 6 | 153032481 | 153033556 | 6q25.2 | 1.075 | 77 | 19686253 | MYCT1 | L1P1_orf2 |
| 7 | 30481972 | 30482979 | 7p14.3 | 1.007 | 9 | NA | NOD1 | L1P1_orf2 |
| 7 | 32390477 | 32392004 | 7p14.3 | 1.527 | 176 | 1907498 | | L1P1_orf2 |
| 7 | 49720296 | 49721702 | 7p12.2 | 1.406 | 7 | 17328292 | | L1P1_orf2 |
| 7 | 49723677 | 49725225 | 7p12.2 | 1.548 | 525 | 1975 | | L1P1_orf2, L1M2_5end, L1P1_5end |
| 7 | 65756060 | 65757315 | 7q11.21 | 1.255 | 41 | 16030835 | TPST1 | L1M2_5end, L1P1_5end |
| 7 | 96479274 | 96481321 | 7q21.3 | 2.047 | 638 | 30721959 | | L1P1_orf2, L1M2_5end, L1P1_5end |
| 7 | 113416172 | 113417537 | 7q31.1 | 1.365 | 367 | 16934851 | | L1P1_orf2 |
| 7 | 141620623 | 141622142 | 7q34 | 1.519 | 419 | 28203086 | | L1P1_orf2 |
| 8 | 18454681 | 18455832 | 8p22 | 1.151 | 153 | NA | PSD3 | Tigger3d,L1PA2_3end |
| 8 | 73787961 | 73789068 | 8q13.3 | 1.107 | 34 | 55332129 | KCNB2 | L1P1_orf2 |
| 8 | 126597079 | 126598414 | 8q24.13 | 1.335 | 237 | 52808011 | | L1P1_orf2 |
| 8 | 126599450 | 126600465 | 8q24.13 | 1.015 | 17 | 1036 | | L1P1_orf2 |
| 8 | 129468131 | 129469267 | 8q24.21 | 1.136 | 138 | 2867666 | | L1P1_orf2 |
| 8 | 129469381 | 129470594 | 8q24.21 | 1.213 | 215 | 114 | | L1M2_5end,L1P1_5end |
| 8 | 135086298 | 135087380 | 8q24.21 | 1.082 | 68 | 5615704 | | L1P1_orf2, L1M2_5end |
| 9 | 96880037 | 96881079 | 9q22.32 | 1.042 | 44 | NA | | L1M2_5end, L1P1_5end |
| 9 | 98464614 | 98465721 | 9q22.32 | 1.107 | 34 | 1583535 | | L1P1_orf2 |
| 9 | 102615779 | 102616803 | 9q31.1 | 1.024 | 26 | 4150058 | NR4A3 | L1P1_orf2 |
| 9 | 135401989 | 135402996 | 9q34.13 | 1.007 | 9 | 32785186 | C9orf171 | L1P1_orf2 |
| 9 | 140912461 | 140914051 | 9q34.3 | 1.590 | 592 | 5509465 | CACNA1B | |
| 10 | 107137274 | 107138381 | 10q25.1 | 1.107 | 34 | NA | | L1P1_orf2 |
| 10 | 111574095 | 111575101 | 10q25.1 | 1.006 | 8 | 4435714 | | L1P1_orf2 |
| 11 | 24351213 | 24352216 | 11p14.3 | 1.003 | 5 | NA | | L1M2_5end, L1P1_orf2 |
| 11 | 24354168 | 24355533 | 11p14.3 | 1.365 | 367 | 1952 | | L1P1_orf2, L1MD_orf2 |
| 11 | 60852454 | 60853848 | 11q12.2 | 1.394 | 373 | 36496921 | | L1P1_orf2 |
| 11 | 85038244 | 85039346 | 11q14.1 | 1.102 | 104 | 24184396 | DLG2 | L1P1_orf2 |
| 11 | 93155588 | 93156702 | 11q21 | 1.114 | 42 | 8116242 | CCDC67 | L1M2_5end, L1P1_orf2 |
| 11 | 93158897 | 93160004 | 11q21 | 1.107 | 34 | 2195 | CCDC67 | L1P1_orf2 |
| 11 | 95170052 | 95171840 | 11q21 | 1.788 | 545 | 2010048 | | L1P1_5end,L1M2_5end,L1P1_orf2 |
| 11 | 95173750 | 95175002 | 11q21 | 1.252 | 204 | 1910 | | L1P1_orf2 |
| 11 | 125410876 | 125411942 | 11q24.2 | 1.066 | 68 | 30235874 | | L1P1_orf2 |
| 12 | 75271094 | 75272762 | 12q21.1 | 1.668 | 318 | NA | | L1P1_orf2 |
| 12 | 88141507 | 88142614 | 12q21.32 | 1.107 | 34 | 12868745 | | L1P1_orf2 |

| | | | | | | | | | |
|---|---|---|---|---|---|---|---|---|---|
| 12 | 126784020 | 126785217 | 12q24.32 | 1.197 | 149 | 38641406 | | L1P1_orf2 | |
| 12 | 126787028 | 126788915 | 12q24.32 | 1.887 | 570 | 1811 | | L1P1_orf2,L1M2_5end, L1P1_5end | |
| 13 | 30220115 | 30221202 | 13q12.3 | 1.087 | 89 | NA | | L1M2_5end, L1P1_5end | |
| 14 | 63587645 | 63588655 | 14q23.2 | 1.010 | 12 | NA | | L1M2_5end | |
| 15 | 55220178 | 55221857 | 15q21.3 | 1.679 | 390 | NA | | L1P1_orf2 | |
| 15 | 71023909 | 71025588 | 15q23.1 | 1.679 | 390 | 15802052 | UACA | L1P1_orf2 | |
| 15 | 83555788 | 83556968 | 15q25.2 | 1.180 | 182 | 12530200 | HOMER2 | L1M2_5end, L1P1_5end | |
| 16 | 236025 | 237244 | 16p13.3 | 1.219 | 200 | NA | | | |
| 16 | 16936305 | 16937984 | 16p12.3 | 1.679 | 390 | 16699061 | | L1P1_orf2 | |
| 16 | 18834487 | 18836369 | 16p12.3 | 1.882 | 489 | 1896503 | SMG1 | L1P1_orf2 | |
| 16 | 83671524 | 83673049 | 16p23.3 | 1.525 | 174 | 64835155 | CDH13 | L1P1_5end, L1M2_5end, L1P1_orf2 | |
| 17 | 64594609 | 64595616 | 17q24.2 | 1.007 | 9 | NA | PRKCA | L1P1_orf2 | |
| 17 | 68457097 | 68458432 | 17q24.3 | 1.335 | 237 | 3861481 | | L1P1_orf2 | |
| 17 | 68459468 | 68461134 | 17q24.3 | 1.666 | 577 | 1036 | | L1P1_orf2 | |
| 18 | 68415980 | 68417774 | 18q22.2 | 1.794 | 319 | NA | | L1P1_orf2 | |
| 19 | 55091938 | 55092944 | 19q13.42 | 1.006 | 8 | NA | LILRA2 | L1PA2_3end, Tigger3d | |
| 22 | 29064027 | 29065134 | 22q12.1 | 1.107 | 34 | NA | TTC28 | L1P1_orf2 | |
| 23 | 11730147 | 11731404 | Xp22.2 | 1.257 | 259 | NA | | | |
| 23 | 11957210 | 11958761 | Xp22.2 | 1.551 | 528 | 225806 | | L1P1_orf2, L1M2_5end, L1P1_5end | |
| 23 | 63474347 | 63475805 | Xq11.2 | 1.458 | 33 | 51515586 | MTMR8 | L1P1_orf2 | |
| 23 | 81101312 | 81102523 | Xq23 | 1.211 | 202 | 17625507 | | L1P1_orf2 | |
| 23 | 114959682 | 114961907 | Xq23 | 2.225 | 1227 | 33857159 | macrosatellite-DXZ4 | TTTA-repeat | +99% |
| 23 | 114962577 | 114964888 | Xq23 | 2.311 | 1313 | 670 | macrosatellite-DXZ4 | TTTA-repeat | +99% |
| 23 | 114965558 | 114967514 | Xq23 | 1.956 | 958 | 670 | macrosatellite-DXZ4 | | +99% |
| 23 | 114968541 | 114969796 | Xq23 | 1.255 | 257 | 1027 | macrosatellite-DXZ4 | | +99% |
| 23 | 114969798 | 114970852 | Xq23 | 1.054 | 56 | 2 | macrosatellite-DXZ4 | TTTA-repeat | +99% |
| 23 | 114971520 | 114973552 | Xq23 | 2.032 | 871 | 668 | macrosatellite-DXZ4 | | +99% |
| 23 | 114974777 | 114976822 | Xq23 | 2.045 | 1047 | 1225 | macrosatellite-DXZ4 | TTTA-repeat | +99% |
| 23 | 114977502 | 114979813 | Xq23 | 2.311 | 1313 | 680 | macrosatellite-DXZ4 | TTTA-repeat | +99% |
| 23 | 114980485 | 114982796 | Xq23 | 2.311 | 1313 | 672 | macrosatellite-DXZ4 | TTTA-repeat | +99% |
| 23 | 114983470 | 114985781 | Xq23 | 2.311 | 1313 | 674 | macrosatellite-DXZ4 | TTTA-repeat | +99% |
| 23 | 114986449 | 114988760 | Xq23 | 2.311 | 1313 | 668 | macrosatellite-DXZ4 | TTTA-repeat | +99% |
| 23 | 114989702 | 114991747 | Xq23 | 2.045 | 1047 | 942 | macrosatellite-DXZ4 | TTTA-repeat | +99% |
| 23 | 114992405 | 114994716 | Xq23 | 2.311 | 1313 | 658 | macrosatellite-DXZ4 | TTTA-repeat | +99% |
| 23 | 114995398 | 114997709 | Xq23 | 2.311 | 1313 | 682 | macrosatellite-DXZ4 | TTTA-repeat | +99% |
| 23 | 114998885 | 115000439 | Xq23 | 1.554 | 224 | 1176 | macrosatellite-DXZ4 | | +99% |
| 23 | 118572124 | 118573459 | Xq24 | 1.335 | 237 | 3571685 | SLC25A43 | L1P1_orf2 | |

| | | | | | | | | | | |
|---|---|---|---|---|---|---|---|---|---|---|
| 23 | 120064672 | 120066902 | Xq24 | 2.230 | 1232 | 1491213 | CT47A-family | AluSc8 | +99% |
| 23 | 120069533 | 120071763 | Xq24 | 2.230 | 1232 | 2631 | CT47A-family | AluSc8 | +99% |
| 23 | 120074394 | 120076624 | Xq24 | 2.230 | 1232 | 2631 | CT47A-family | AluSc8 | +99% |
| 23 | 120079254 | 120081484 | Xq24 | 2.230 | 1232 | 2630 | CT47A-family | AluSc8 | +99% |
| 23 | 120084115 | 120086345 | Xq24 | 2.230 | 1232 | 2631 | CT47A-family | AluSc8 | +99% |
| 23 | 120088975 | 120090501 | Xq24 | 1.526 | 351 | 2630 | CT47A-family | | +99% |
| 23 | 120094352 | 120096088 | Xq24 | 1.736 | 707 | 3851 | CT47A-family | AluSc8 | +99% |
| 23 | 120098719 | 120100949 | Xq24 | 2.230 | 1232 | 2631 | CT47A-family | AluSc8 | +99% |
| 23 | 120103579 | 120105809 | Xq24 | 2.230 | 1232 | 2630 | CT47A-family | AluSc8 | +99% |
| 23 | 120108439 | 120110669 | Xq24 | 2.230 | 1232 | 2630 | CT47A-family | AluSc8 | +99% |
| 23 | 120113299 | 120115529 | Xq24 | 2.230 | 1232 | 2630 | CT47A-family | AluSc8 | +99% |
| 23 | 120118159 | 120120389 | Xq24 | 2.230 | 1232 | 2630 | CT47A-family | AluSc8 | +99% |
